# The reproducibility of MTV and TLG of soft tissue tumors calculated by FDG-PET: Comparison between the lower limit by the fixed value SUV 2.5 and that value by 30% of SUVmax

**DOI:** 10.1007/s11604-022-01378-8

**Published:** 2023-01-13

**Authors:** Hitomi Iwasa, Shigeki Nagamachi, Shizuhide Nakayama, Takuaki Yamamoto, Kengo Yoshimitsu

**Affiliations:** 1 Department of Radiology, Faculty of Medicine, Fukuoka University, 7-45-1 Nanakuma, Jonan-Ku, Fukuoka, 814-0180 Japan; 2 Department of Diagnostic and Interventional Radiology, Kochi Medical School, Kochi University, Kochi, Japan; 3 Department of Orthopedic Surgery, Faculty of Medicine, Fukuoka University, Fukuoka, Japan

**Keywords:** Soft-tissue tumor, MTV, TLG, FDG-PET, Reproducibility

## Abstract

**Purpose:**

We evaluated the reproducibility calculating volume-based FDG-PET/CT parameters, i.e., metabolic tumor volume (MTV) and total lesion glycolysis (TLG), in soft tissue tumors.

**Materials and methods:**

Fifty-three cases with soft tissue tumors were analyzed retrospectively. The conditions determining the lower limit of MTV were fixed value SUV 2.5 or 30% of SUVmax. To investigate the agreement of the measurements by two radiologists, %difference, the correlation coefficients and Bland–Altman plot were analyzed. We compared these parameters in both intra- and inter-operator for evaluating the agreement in the measurements.

**Results:**

The values of % difference were excellent, 0.2–3.5%, in the intra-operator in all calculated volume-based parameters. In both inter- and intra-operator analysis, the values of % differences were lower in the parameters calculated by SUV 2.5 fixed value as a lower threshold compared with those calculated by 30% of SUVmax as a lower threshold. The correlation coefficient in MTV30% for inter-operator were 0.84 or 0.87, those were lower than values by the intra-operator evaluation. Nevertheless, the correlation coefficients were higher than 0.84 in every parameter. Particularly, correlation coefficient in the parameters calculated by SUV 2.5 fixed value was better than those calculated by 30% of SUVmax. The Bland–Altman plot analysis showed good agreement for all parameters, particularly in the intra-operator examinations. However, in the inter-operator study, some variances were noted in every condition.

**Conclusion:**

In conclusion, the reproducibility of measuring volume-based FDG-PET/CT parameters of soft tissue tumors was good, particularly, in the measurement by fixed lower limit value SUV 2.5 in the intra-operator.

## Introduction

Positron emission tomography (PET) using ^18^F-fluorodeoxyglucose (FDG) is used as a useful imaging examination for the diagnosis of soft-tissue malignant tumors, prognostic evaluation, and evaluation of therapeutic effects [1]. As the tumor accumulation indices, single voxel parameters such as the maximum of the standardized uptake value (SUVmax) and the peak of the SUV (SUVpeak), and the volume-based parameters such as metabolic tumor volume (MTV) and total lesion glycolysis (TLG) are used.

With regard to the usefulness of the volume-based parameters using FDG-PET/CT in soft-tissue tumors, there are reports that it correlates with tumor proliferation potential [2] and that it is more useful than single voxel parameters such as SUVmax for the prognostic evaluation [3–6]. In recent, so-called precision medicine is promoted as tailor-made therapy [7]. Volume-based parameter may contribute to futures precision medicine because of one of the determinants for prognosis [8].

Furthermore, it has been reported that the therapeutic result based on PET Response Criteria in Solid Tumors (PERCIST) criteria are also useful in stratifying prognosis [9]. In PERCIST, the decision based on changes in TLG of the volume-based parameters will be preferred over changes in SUVpeak [10].

For the calculation of volume-based parameters, drawing of the tumor contour is required. Various methods for automatically tumor demarcation by FDG-PET have been devised and used, such as the absolute SUV threshold method [11], the fixed% SUVmax threshold method [11, 12], and extracting the tumor contour using special software [13]. However, the standardized best method for the tumor metrics by FDG-PET/CT have not been determined yet, due to the several problems such as intra-tumoral heterogeneity of sarcomas [6].

In addition, the reproducibility for calculating these indexes is very important issues. Regarding the reproducibility of parameter measurement in primary lung cancer, the method using fixed the lower threshold by the certain SUV value is known to have the best reproducibility [11]. Comparing to chest region, both upper and lower limbs are free from influence by respiratory motion. The tumor contour of soft tissues in these areas, therefore, could be clearly demarcated and the reproducibility for calculating PET parameters could be better than that in other organs.

Yet, to the best of our knowledge, there have been no reports on the reproducibility in calculating volume-based parameters, despite many reports of usefulness using the volume-based parameters for soft tissue tumors [2, 3, 9, 14]. It is necessary to clarify this issue when using these volume-based parameters in actual clinical practice. If the issue is clarified, it will also be helpful for assisting calculation of volume-based parameters using new techniques such as artificial intelligence (Al).

In this study, we retrospectively examined the reproducibility for calculation of volume-based parameters in preoperative FDG-PET/CT of soft-tissue tumors with a confirmed pathological diagnosis after operation. For the study, we chose both 30% of SUVmax and SUV 2.5 as the lower thresholds which was used in the previous study [11].

## Methods

### Subjects

The subjects were 53 patients who underwent FDG-PET/CT before resection for soft-tissue tumors at Fukuoka University Hospital between June 2010 and May 2021 (Table1).

**Table 1:** Lists of the histopathology in the analyzed cases

Tumor histology	Patients (*n* = 53)
Myxofibrosarcoma	20
Liposarcoma	6
Malignant lymphoma	4
Leiomyosarcoma	3
Rhabdomyosarcoma	3
Undifferentiated pleomorphic sarcoma	3
Synovial sarcoma	2
Epithelioid sarcoma	2
Alveolar soft part sarcoma	2
Myxoinflammatory fibroblastic sarcoma	1
Extraosseous osteosarcoma	1
Malignant melanoma	1
Giant cell tumor	1
Schwannoma	1
Desmoid type fibromatosis	1
Solitary fibrous tumor	1
Superficial angiomyxoma	1

Among them, 30 were males and 23 were females. The mean age of the patients was 61.5 ± 19.1 years (range 17–91 years). Except for three cases (schwannoma, desmoid type fibromatosis, and superficial angiomyxoma), all examined cases were malignant. The tumor located areas were the lower extremities in 35 cases, the upper extremities in 14 cases, the head and neck in 2 cases, and the thorax in 2 cases.

### PET/CT examination

All patients had 5 h fasting (only plain water was allowed) and a fasting blood sugar was less than 150 mg/dl before receiving an intravenous 18F-FDG dose of 3 MBq/kg. During uptake period (60 min) patients were requested to lie comfortably and allowed to take about 500 ml of plain water. PET/CT imaging was done with Aquiduo (Canon Co, Japan) with a 16-slice Light-Speed CT component. A low-dose CT examination from head to toe followed by acquisition of PET imaging using 3 min/bed position from toe to head was conducted in all patients. The PET data were reconstructed using a CT transmission map for attenuation correction with the ordered-subsets expectation maximization (OSEM) algorithm (4 iterations, 14 subsets) and a Gaussian Filter (FWHM = 7.0 mm) and displayed in a 128 matrix (pixel size = 3.9 × 3.9 mm with a slice thickness of 2.0 mm).

### Data analysis

The analysis software was GI-PET (Version1. AZE VirtualPlace, Falcon). GI-PET is one of softwares contained in the software package working on the personal computer (PC) and is easily used. Even researchers without special workstation would be able to measure volumetric parameters with PC and GI-PET. Because of such reason, we used GI-PET in the present study. For proceeding the analysis procedure, the DICOM data including PET data and CT data transferred to GI program through portable recording medium.

In the GI program, whole body PET/CT images were reconstructed automatically. On the 3D PET/CT images, that is sagittal, axial, and coronal plane, spherical VOIs including the tumor uptake were manually set by the two operators, respectively. If the uptake was inhomogeneous such as tumor combined with solid and cystic component, the spherical VOI also including tumor component without FDG uptake was set by referencing both PET/CT and CT.

Two operators measured retrospectively MTV and TLG in the two conditions, namely, the lower limit by the fixed value SUV 2.5 and that value by 30% of SUVmax. Both MTV2.5 and TLG2.5 were the parameters determined using the absolute SUV threshold method. Both MTV30% and TLG30% were the parameters determined using the fixed % SUVmax threshold method. Both values of MTV30% and TLG30% were measured in all 53 cases, while the values of both MTV2.5 and TLG2.5 were measured in 43 cases because 10 cases had the value of SUVmax was less than 2.5. The operators were a radiologist with more than 30 years of experience in nuclear medicine (operator 1) and a radiologist with 8 years of experience in nuclear medicine (operator 2).

Evaluation methods were as follows: (1) comparison of the mean values of each volume-based parameters among two operators; (2) comparison of the inter-operator reproducibility between the operator 1 and the operator 2, and (3) comparison of the intra-operator reproducibility within the operator 2, who performed the examinations at 1-week intervals. That is, the operator 2 measured twice (operator 2-1 and operator 2-2). The study parameters were ①inter- and intra-operator %difference comparisons for MTV30%, MTV2.5, TLG30%, and TLG2.5; ② correlation coefficient, and ③ Bland–Altman plot analysis, respectively.

Paired t test was applied in the comparison of mean values of MTV2.5, MTV30, TLG2.5 and MTV30 among operators. In addition, paired t test was done for comparison of parameters among every volume-based parameter for analyzing intra- and inter-reproducibility. The correlation analysis was done by Poisson correlation analysis. The statistically significance was determined to be significant with a *P* < 0.05. The used software was Graphpad Prism 8 (MDF. Ltd. Tokyo. Japan).

### Ethics

This retrospective study was approved by the Ethics Committee of the Faculty of Medicine, Fukuoka University, with ethical number U20-08-003. The procedures performed in the present study were performed in accordance with the ethical standards of the institutional and/or national research committee and with the 1964 Helsinki declaration or comparable ethical standards.

## Results

In the comparison of the volume-based parameters among two operators, there was no statistical significance, when we analyze 43 cases whose SUVmax is higher than 2.5 (Table 2). While, in the analysis of 53 cases including patient whose SUVmax is lower than 2.5, both values of MTV30% and TLG30% were significantly lower in the operator 1 (Table 3).

**Table 2:**
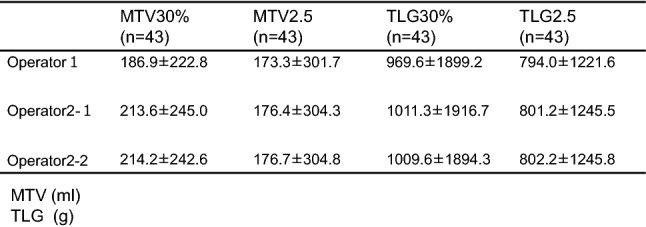
Measurements of MTV and TLG

**Table 3:**
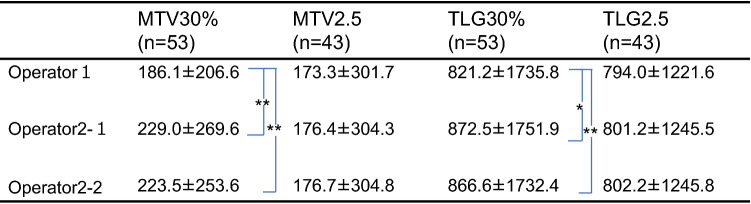
Measurements of MTV and TLG

MTV (ml) TLG (g)

*P < 0.01

**P < 0.05

The values of inter-operator %difference between operator 1 and operator 2-1 was 33.5% for MTV30%, 6.0% for MTV2.5, 21.9% for TLG30%, and 3.3% for TLG2.5. The values of inter-operator %difference between operator 1 and operator 2-2 was 32%, 5.9%, 21.1%, and 3.2%, respectively, and the values of intra-operator %difference between operator 2-1 and operator 2-2 was 3.1%, 0.3%, 2.5%, and 0.2%, respectively. The values of %difference of each parameter for MTV30%, TLG30%, and TLG2.5 was significantly lower in the intra-operator compared to those in inter-operator. On the other hand, there was no significant difference between inter- and intra-operator for MTV2.5. In the comparison of inter-operator %difference, both TLG30% and TLG2.5, showed significantly lower values compared with MTV30% and MTV2.5, respectively. Particularly, TLG2.5 showed the lowest value. Among % differences intra-operator comparison, TLG2.5 also showed the significantly lowest value (Tables 4, 5).

**Table 4:**
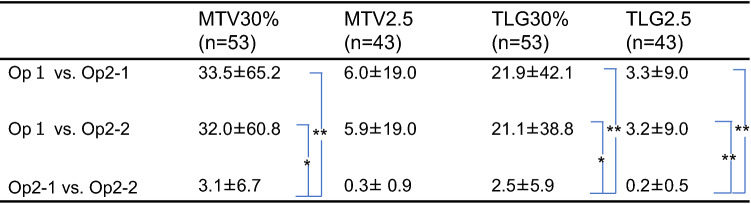
% Difference (comparison within parameter)

Unit (%)

*P < 0.01

**P < 0.05

**Table 5:**
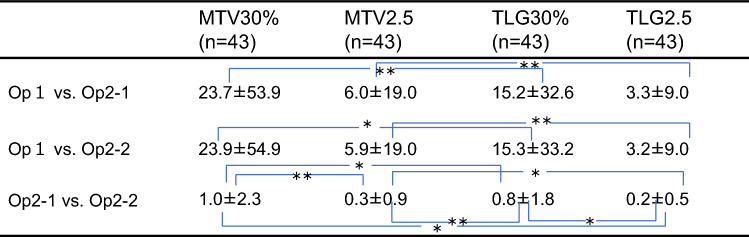
% Difference (comparison between parameter)

Unit (%)

*P < 0.01

**P < 0.05

The value of the correlation coefficient for inter-operator was 0.84–0.87 in MTV30%, lower than the value 0.99 for intra-operator. The inter- and intra-operator correlation coefficients were 0.99 or higher for MTV2.5, TLG30%, and TLG2.5 (Table 6).

**Table 6:** Comparison of correlation coefficient

	MTV30% (*n* = 53)	MTV2.5 (*n* = 43)	TLG30% (*n* = 53)	TLG2.5 (*n* = 43)
Op1 vs. Op2-1	0.84	0.99	0.99	0.99
Op1 vs. Op2-2	0.87	0.99	0.99	0.99
Op2-1 vs. Op2-2	0.99	1.00	0.99	1.00

*P* < 0.0001

**Fig. 1:**
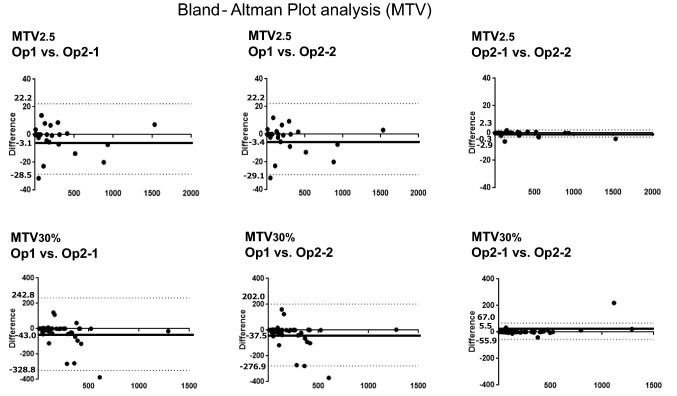
Bland–Altman plot analysis of inter- and intra-operator reproducibility in MTV. Both MTV2.5 and MTV30% showed good agreement inter- and intra-operators, especially intra-operators

**Fig. 2:**
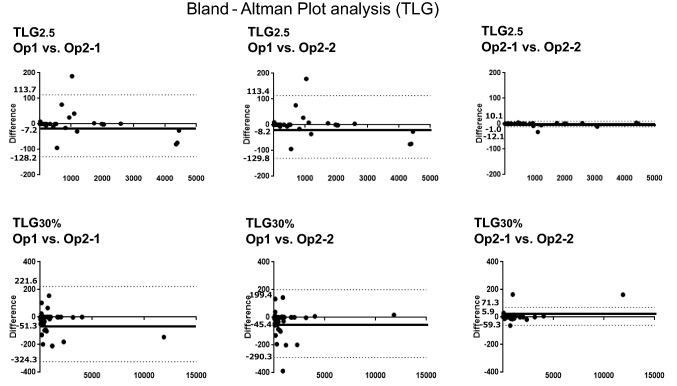
Bland–Altman plot analysis of inter- and intra-operator reproducibility in TLG. Both TLG2.5 and TLG30% showed good agreement inter- and intra-operators, especially intra-operators

In the Bland–Altman plot analysis of inter-operator evaluation, the ranges of the 95% limit of agreement for MTV2.5 were narrower than those for MTV30%. Similarly, those ranges for TLG2.5 were narrower than those for MTV30%. In the intra-operator analysis, they showed similar tendency, these ranges of the 95% limit of agreement for MTV2.5 and TLG2.5 showed narrower values compared to those with MTV30% or TLG30% (Figs. 1, 2).

### Case presentation

**Fig. 3:**
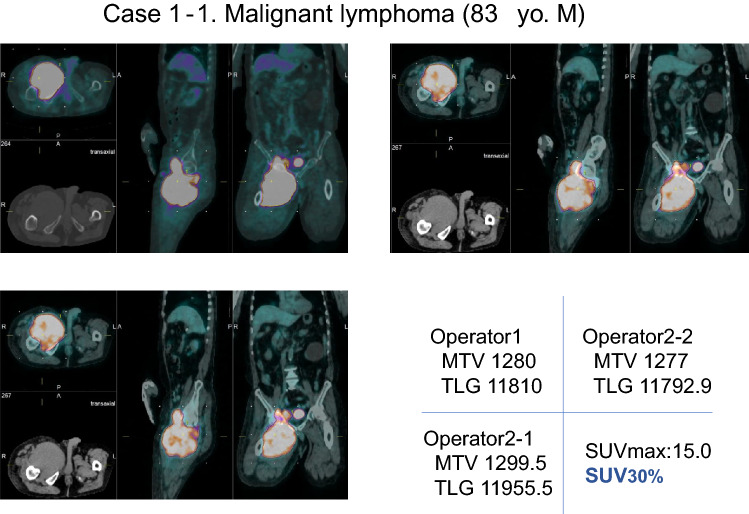
An 83-year-old male with a large soft-tissue malignant lymphoma in the right inguinal region to thigh. There were variabilities in MTV30% and TLG30% among inter- and intra-operators

**Fig. 4:**
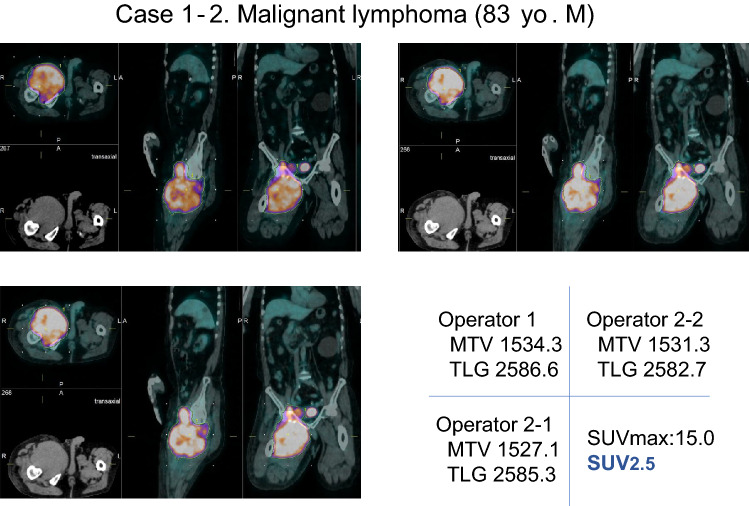
When the lower limit by the fixed value SUV2.5 was used, the variability of MTV and TLG among inter- and intra-operators was smaller than cases 30% value of SUVmax was used as lower limit

Case 1 (Figs. 3, 4).

An 83-year-old male, FDG-PET/CT demonstrates large mass in the right inguinal region to the right thigh with intense FDG uptake. Post-operative histopathological diagnosis was malignant lymphoma (diffuse large cell B cell lymphoma, DLBCL). The agreement of the SUV30% values were not bad, MTV30% 1280 and TLG30% 11810 by operator 1, MTV30% 1299.5 and TLG30% 11955.5 by operator 2-1, and MTV30% 1277 and TLG30% 11792.9 by operator 2-2. As for SUV2.5 values, they also showed relatively good agreement. namely, MTV2.5 1534.3 and TLG2.5 2586.6 by the operator 1, MTV2.5 1527.1 and TLG2.5 2585.3 by the operator 2-1, MTV2.5 1531.3 and TLG2.5 2582.7 by the operator 2-2.

**Fig. 5:**
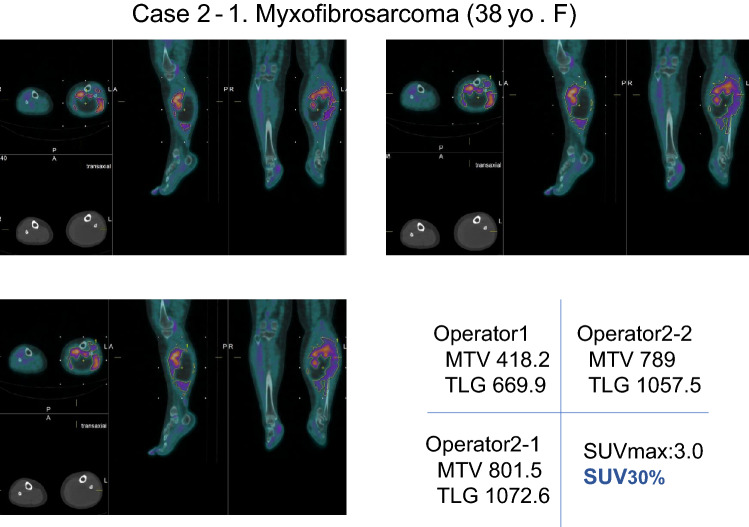
A 38-year-old female with a soft-tissue tumor in the left lower leg; histopathology result was myxofibrosarcoma. When 30% value of SUVmax as lower limit was used, there was variability in MTV and TLG among inter- and intra-operators

**Fig. 6:**
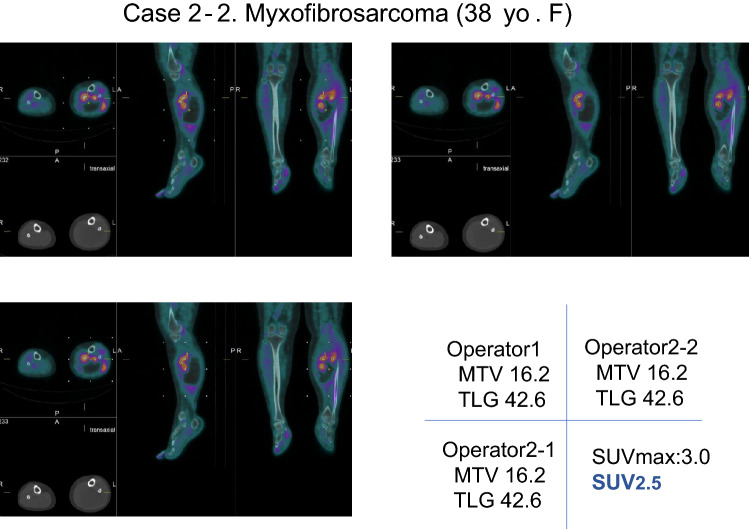
When the lower limit by the fixed value SUV 2.5 was used, the inter- and intra-operators MTV and TLG measurements were accorded

Case 2 (Figs. 5, 6).

A 38-year-old female. FDG-PET/CT shows soft-tissue tumor in the left lower leg with high FDG uptake. Post-surgical histopathology result was myxofibrosarcoma. Regarding the values SUV30%, the values of operator 1 were an MTV of 418.2 and a TLG of 669.9. The values of first data of operator 2 were an MTV of 801.5 and a TLG of 1072.6, and the second data of operator 2 were an MTV of 789 and a TLG of 1057.5. Several variations of measured values were observed inter and intra examiners, respectively. On the other hand, the value of SUV2.5 was well accorded in both operators, namely 16.2 in MTV and 42.6 in TLG by operator 1. Same values were observed in both the first and the second of operator 2’s data.

## Discussion

In this study, we confirmed the good reproducibility in the measurement of volume-based parameters, particularly in intra-operator conditions. Although some reports have used by fixed threshold SUV2.0 for determining the lower threshold limit [2, 15], we adopted SUV2.5, which is commonly used in many tumors [15, 16].

In addition, we also evaluated the MTV measuring condition by certain % of SUVmax. Although Anderson et al. them as 40% of SUVmax [3], it was not able to cover entire tumor volume in cases with containing FDG low uptake component. Therefore, we analyzed volume-based parameters calculated by lower threshold limit 30% of SUVmax.

As a result, the correlation coefficients were generally excellent between every two conditions, although those inter-observer values in MTV30% were relatively lower than those of other conditions. The values of % differences in inter-operators for the volume-based parameters were less than 6% when we use SUV2.5, which was better than the %difference of about 15–35% in MTV30% or TLG30%. According to Bland–Altman plot analysis, tumors with lower value MTV or TLG, the variabilities tended to be larger in every inter-observer comparison conditions.

In general, for calculating volume-based PET parameters, the values vary according to the image reconstruction method, imaging workstation, software, examination protocol [17–20]. Yet, in the series of the present study, the analysis was done on the same data by the two operators retrospectively. Both operators used the same software, same version. Namely, all process by each operator was same until just before VOI setting. Therefore, we focused on the operator-dependent factor in spherical VOI setting around the tumor, because software-dependent factor nor image construction method, examination protocol seemed be unrelated.

One possible factor of the less predominance of reproducibility in SUV30% was the inter-operator variability in the spherical VOI setting covering entire tumor contour along avoiding physiological high uptake by urinary bladder. At first, each operator sets larger spherical VOI including tumor manually. During this procedure, spherical VOI’s location and size may be different by operator. Namely, when setting a spherical VOI including tumors that extend from near the thighs to the pelvic region, the VOI setting will be different to avoid physiological FDG high uptake such as urinary bladder or large intestine. Although each tumor contours 30% area or SUV2.5 areas are automatically drawn within the spherical VOI, peritumor non-tumor components with high uptake such as part of urinary bladder, large intestine, or other soft tissues next to tumor could also be contained variously. Those processes could have influenced the variability in the measurement. Both MTV30 and TLG30 likely to be more influenced compared to those by fixed threshold SUV2.5 because non-tumor components might be more included in the conditions of MTV30 and TLG30. Therefore, in case such as case1, the tumor located adjacent to high physiological uptake, the reproducibility of the calculation would be worse than soft tissue tumor in areas without physiological high uptake, particularly in both MTV30 and TLG30.

The other influential factors were tumor shape and its component, the ratio of cystic and solid component. In setting spherical VOI around tumors with peripherally existed cystic component, the VOI setting should outside of the cystic component. In this procedure, some differences in spherical VOI size will be noted dependent on operator’s technique. Even if cystic lesions were not included in the automatically drawn tumor VOI due to its low uptake, its wall and para wall non-tumor component may show variable uptake and can be included in tumor VOI. Accordingly, automatically drawn tumor VOI should be larger, when operators set the first spherical VOI larger size including marginal non-tumor component. Thus, in the analysis for patient such as case 2, heterogeneous distribution of solid and cystic component and the size of operator's set spherical VOI might have influenced the measurement reproducibility.

Conceivable methods to overcoming these causative factors for variability are as follows. The one method is removal of the physiologic high uptake by urination or defecation just before PET/CT examination. Second, a spherical VOI should be set as close to the tumor as possible to minimize the operator’s dependent variability.

The limitation of the studies is as follows. Because of the retrospective study, examined cases include relatively uncommon diseases. Whether the preoperative PET examination should be done or not is determined by the patients’ doctors. According to the Clinical Practice Guidelines 2020 for the Management of Soft Tissue Tumors by Japanese Orthopedic Association (JOA), lung is the most frequently metastatic organ from the soft tissue sarcoma. The similar research has been reported [21]. Therefore, chest CT is routinely selected for preoperative examination. On the other hand, although FDG-PET/CT is excellent equipment for screening the distant metastasis, its routine use is limited. As it does not so impact for changing the method of therapy [1], FDG-PET/CT was not always chosen in our institute. Such a background might have influenced the bias of kinds of the diseases.

Finally, the present study demonstrated that the measurement of volume-based parameters under the condition with lower limit SUVmax 2.5 showed better reproducibility compared with that of lower limit 30% of SUVmax. The result was similar with previous study for lung cancer, namely, the measurement by threshold with fixed values showed higher reproducibility compared with that by threshold of certain % value of SUVmax [11]. In future, if the volume-based parameter is involved in therapeutic procedure, fixed value such as SUVmax 2.5 should be used for calculating MTV or TLG.

## Conclusion

In the calculation of volume-based parameters of soft-tissue tumors on FDG-PET/CT, the reproducibility was better in conditions of MTV2.5, TLG2.5 compared with those of MTV30%, and TLG30%, respectively. Particularly, the better reproducibility was confirmed in intra-operator conditions.
